# Efficient assessment of brain fog and fatigue: Development of the Fatigue and Altered Cognition Scale (FACs)

**DOI:** 10.1371/journal.pone.0295593

**Published:** 2023-12-11

**Authors:** Timothy R. Elliott, Yu-Yu Hsiao, Kathleen Randolph, Randall J. Urban, Melinda Sheffield-Moore, Richard B. Pyles, Brent E. Masel, Tamara Wexler, Traver J. Wright

**Affiliations:** 1 Department of Educational Psychology, Texas A&M University, College Station, Texas, United States of America; 2 Department of Individual, Family, and Community Education, University of New Mexico, Albuquerque, New Mexico, United States of America; 3 Department of Internal Medicine, University of Texas Medical Branch, Galveston, Texas, United States of America; 4 Department of Pediatrics, University of Texas Medical Branch, Galveston, Texas, United States of America; 5 Department of Neurology, University of Texas Medical Branch, Galveston, TX, United States of America; 6 Department of Rehabilitation Medicine, New York University Langone Health, New York, NY, United States of America; St John’s University, UNITED STATES

## Abstract

Debilitating symptoms of fatigue and accompanying “brain fog” are observed among patients with various chronic health conditions. Unfortunately, an efficient and psychometrically sound instrument to assess these co-occurring symptoms is unavailable. Here, we report the development and initial psychometric properties of the Fatigue and Altered Cognition Scale (the FACs), a measure of self-reported central fatigue and brain fog. Traumatic brain injury (TBI) was chosen to model and develop the FACs due to research team expertise and established links between TBI and the symptom complex. Potential items were generated by researchers and clinicians with experience treating these symptoms, drawing from relevant literature and review of patient responses to measures from past and current TBI studies. The 20 candidate items for the FACs—ten each to assess altered cognition (i.e., brain fog) and central fatigue–were formatted on an electronic visual analogue response scale (eVAS) via an online survey. Demographic information and history of TBI were obtained. A total of 519 participants consented and provided usable data (average age = 40.23 years; 73% female), 204 of whom self-reported a history of TBI (75% reported mild TBI). Internal consistency and reliability values were calculated. Confirmatory factor analysis (CFA) examined the presumed two-factor structure of the FACs and a one-factor solution for comparison. A measurement invariance test of the two latent constructs (altered cognition, fatigue) among participants with and without TBI was conducted. All items demonstrated normal distribution. Cronbach’s alpha coefficients indicated good internal consistency for both factors (α’s = .95). Omega reliability values were favorable (α’s = .95). CFA supported the presumed two-factor model and item loadings which outperformed the one-factor model. Measurement invariance found the two-factor structure was consistent between the two groups. Implications of these findings, study limitations, and potential use of the FACs in clinical research and practice are discussed.

## Introduction

Co-occurring symptoms of fatigue and brain fog result in a debilitating symptom complex with a strikingly similar manifestation in over a dozen different chronic conditions [1] including traumatic brain injury (TBI) [2, 3], chronic fatigue syndrome [4], hypoparathyroidism [5], celiac disease [6], postural tachycardia syndrome [7], fibromyalgia, and rheumatoid arthritis [8]. Brain fog and fatigue are also considered cardinal symptoms of post-acute sequelae of COVID-19 (PASC) [9, 10]. Among persons with TBI, this symptom cluster is recognized as an element of post concussive syndrome [11] that persists over time [3, 12, 13], with debilitating effects on rehabilitation efforts [14], community reintegration [15], and clinical treatment and monitoring [2, 16].

Fatigue and brain fog are terms frequently used without clear definitions, compromising our understanding, assessment, and treatment of these symptoms. Fatigue associated with these chronic health conditions is complex, and distinctions are not typically made between *peripheral* and *central* fatigue [17]. Peripheral fatigue refers to muscular impairment or exhaustion due to exertion; central fatigue, however, is a subjective report of an individual’s difficulty initiating and maintaining activity and attend to tasks that require sustained mental effort [17]. Brain fog is a colloquial term frequently used by patients and clinicians to describe subjective difficulties with thinking and focus, forgetfulness, lack of mental clarity, slow conversational reaction times, and occasional problems with word selection [2, 12, 18]. Studies using various neuropsychological instruments indicate that brain fog reflects deficits in attention, short-term and working memory, processing speed, and concentration [4, 19]. Nevertheless, there is no real consensus on the definition of brain fog and despite this, the Centers of Disease Control includes it as a symptom of mild TBI and concussion [20].

The frequent co-occurrence of these symptoms among patients with various chronic health conditions–and the difficulty in diagnosing and treating them–has stimulated interest in possible common pathways that might underpin their development and expression. Because traumatic brain injuries (TBIs) are common in the population and have an established link to this fatigue and altered cognition (FAC) symptom cluster, individuals with a history of TBI provide an opportune model to study this condition. A conceptualization that informs the current study focuses on problems with pituitary dysfunction and reduced growth hormone (GH) secretion that are commonly observed following TBI [2, 21–23]. The symptom cluster is well-described in patients who experience deficiencies in pituitary hormones including growth hormone, the most frequent deficiency identified post-TBI [24]. The complex interplay between the nature of the TBI and GH dysfunction contributes to patient symptoms of central fatigue and altered cognition consistent with our understanding of brain fog [23]. This syndrome has been named Brain Injury Associated Fatigue and Altered Cognition (BIAFAC) [2], and evidence indicates that GH replacement therapy effectively reduces these symptoms [2, 25, 26]. The fatigue associated with BIAFAC is often profound and unrelenting, leaving patients unable to maintain jobs or activities of daily living. The cognitive dysfunction includes short term memory loss, delayed processing speed and word selection, and problems with executive function. Although GH treatment greatly reduces symptom severity, symptoms of fatigue and cognitive impairment do not resolve on the same timeline. Preliminary clinical work indicates that fatigue symptoms improve approximately 3 months after starting GH therapy, and altered cognition (i.e., brain fog) begins to clear after 4 or 5 months of treatment. Moreover, when GH treatment is stopped, fatigue typically returns in 3 months and cognitive dysfunction in 4 to 5 months [2].

An instrument that directly and efficiently assesses patient reports of central fatigue and brain fog is needed to advance our understanding of this symptom cluster. For example, no single specific instrument exists to measure brain fog, and some studies of brain fog utilize over a dozen neuropsychological measures to isolate these deficits [4, 19, 22]. Using multiple overlapping instruments is cumbersome, time-intensive, and expensive for diagnostic purposes and for clinicians to monitor symptom response to treatment. Many cognitive tests commonly used to assess severe neuropsychological impairments are not sensitive to the experience of and changes in brain fog reported by patients [22]. Using multiple overlapping instruments fatigues the patient (which can adversely affect test results) and reduces statistical power for researchers monitoring symptoms when adjusting for multiple testing. Psychometrically sound instruments that are sensitive to potential changes in symptoms and can be used in an efficient and timely manner are preferred in clinical practice and research [27]. A versatile and specific tool is needed to expedite clinical diagnosis and symptom monitoring for treatment and research purposes.

### The present study

The primary objective of the present study was to develop and test an efficient and specific instrument to assess the presence and severity of the FAC symptom cluster. Meeting this objective would provide the field with a sensitive, reliable tool for use in clinical research and practice to inform clinical decision-making and monitor response to treatment. Ideally, the instrument could also be used for patients with other health conditions that also manifest with the FAC symptom cluster. Although other measures of fatigue are currently available, the instrument described in this study focuses on symptoms of central fatigue co-occurring with brain fog that may share an underlying common pathway that is responsive to clinical intervention.

The instrument we describe in this study was designed for use across various digital platforms (e.g., computer tablets, smart phones, laptop computers) to expedite its application in clinical practice and research. The digitized instrument was designed for easy distribution and clinical use with patients, conveniently administered by health care providers. This format also features automated scoring to rapidly provide preliminary normative data and serve as a sensitive tool necessary to monitor longitudinal change in symptom severity for both clinical and research purposes.

In this paper we report the development and preliminary validation of a questionnaire to efficiently assess the presence and severity of co-occurring brain fog and fatigue as described in the BIAFAC model. We describe the item selection of the Fatigue and Altered Cognition Scale (the FACs), and its unique format designed to maximize utility in clinical research and practice. We report results from a confirmatory factor analysis of the items, administered in a web-based survey to respondents with and without a self-reported history of TBI. We also conduct a comparative test between the proposed two-factor model with a one-factor model and examine the measurement invariance of the instruments and its items between participants with and without a history of TBI.

## Materials and methods

### Ethics statement

The study was conducted in accordance with the principles of the Declaration of Helsinki and was approved by both the Texas A&M Institutional Review Board (IRB #2021-0836D) and the University of Texas Medical Branch Institutional Review Board (IRB #21–0182). This anonymous online study was deemed minimal risk and granted a waiver of written informed consent. All methods adhered to relevant guidelines and regulations.

### Sample

Prospective participants were recruited using social media, university listservs, and researchmatch.org. ResearchMatch is a national health volunteer registry that was created by several academic institutions and supported by the U.S. National Institutes of Health as part of the Clinical Translational Science Award program. ResearchMatch has a large population of volunteers who have consented to be contacted by researchers about health studies for which they may be eligible.

The invitation to participate stated that the study was “seeking male and female volunteers between the ages of 18 and 70 to take part in an online research questionnaire examining the effects of traumatic brain injury (TBI). Volunteers with and without a history of TBI are needed.” Prospective participants were informed the questionnaire should take between 10 and 15 minutes to complete. Individuals interested in the study had to email the study coordinator to obtain a link to the questionnaire. Participants that responded with interest in the study were provided a link for digital online screening and completion of the questionnaire. The digital platform was supported and hosted by Texas A&M University using Qualtrics online software (Qualtrics International Inc. Seattle, WA, USA). Respondents could participate using compatible digital devices including laptop computer, tablet, and smart phone.

The initial screen of the survey provided a description of the study, contact information for the lead investigators and institutional review boards, and details about confidentiality, potential harm, and the option to leave the study at any time. Prospective participants were asked if they wished to participate in the study and indicated consent by marking “I agree” or “I disagree.” Participant anonymity was maintained, and they were not asked to provide any contact information. Participants were informed they could print a copy of the consent form from their computer screen. Respondents were stratified into TBI and non-TBI groups based on their response to an initial question of “Have you had a TBI/concussion?”

### Measures

The study survey collected basic demographic information from qualifying participants and included two instruments relevant to the present study including the FACs and an additional instrument to collect past TBI experience (TBI participants only).

#### Past TBI experience

Individuals who reported a history of a TBI or concussion were prompted to complete the Ohio State University TBI Identification Method (OSU TBI-ID) [28]. The OSU TBI-ID is an established instrument to determine lifetime history of TBI, based on case definitions provided by the Centers for Disease Control [29]. It is a recommended core data element for assessing lifetime incidence of TBI (History of Disease/Injury Event) [30], and it has been effectively used in online surveys [31]. The measure asks individuals to report an experience of a TBI in their lifetime, including details about the cause of the TBI, their age at time of injury, and presence and length of loss of consciousness (LOC). Subsequent items clarify the severity of the TBI (mild, moderate, and severe) and details about past TBI experiences.

#### Development of the Fatigue and Altered Cognition Scale (FACs)

To develop items for the FACs instruments, potential items were generated by members of the research team based on clinical experience, prior research, and the relevant literature. The team included a physician with over 20 years of treating BIAFAC patients with GH therapy. Initial item selection was informed by an analysis of responses to measures used in prior [21, 22] and ongoing TBI-related studies conducted by the research team. Relevant items were considered if they demonstrated positive response to treatment. These were evaluated and prioritized by the research team based on the degree to which these items (a) were consistent with the working BIAFAC descriptions of central fatigue and brain fog, and (b) were consistent with patient reports while receiving clinical care [2]. Potential items were further refined for simple, concise, and consistent wording across the questionnaire while capturing the symptoms commonly reported by patients treated for BIAFAC.

The initial list included 20 items, ten each for the assessment of brain fog (labeled as “altered cognition”) and fatigue. A reporting time frame of 2 weeks was selected to balance the need for sensitivity to capture changes in symptom severity over time (e.g., with treatment) while buffering short-term daily volatility in mood. To facilitate the use of the instrument in clinical research and practice, we based the response set on an “electronic” visual analogue rating (eVAS) scale recognized for its ease in administration and scoring [32, 33]. A recent review concludes that the eVAS format is equivalent to the use of paper VAS, and acceptable for clinical practice [34]. Each item was anchored with descriptors indicative of extreme responses (“*not at all*” to “*extremely*”). For use with computer devices that may be used in clinical interactions and in research projects (e.g., laptop, tablet), responses to each item were made with a “drag and drop” slider bar. Participants moved the slider along a horizontal line to denote their response. Consistent with contemporary practice, the response was set proportionally to obtain a 0 to 100 score for each item [34]. The pilot questionnaire was formatted for online administration using the Qualtrics platform.

### Statistical analyses

Descriptive statistics were calculated for both fatigue and cognition items. Responses distribution for each item were further evaluated. Ideally, the item response categories (i.e., 0 to 100) should be fully utilized by participants, and the items should include a wide range of fatigue and cognition levels among participants. Next, we estimated the reliability of the scale scores in the FACs. Internal consistency was determined with Cronbach’s alpha [35] and the composite reliability values were calculated with the omega composite [36, 37].

The construct validity of the FACs was examined using confirmatory factor analysis (CFA). CFA is the preferred approach when a conceptual model is used to guide the construction and analysis of items selected to measure theoretical constructs as described in a conceptual model [27, 38]. Consequently, we were primarily interested in testing a two-factor model consistent with the BIAFAC conceptualization of fatigue and brain fog. We specified a two-factor model in which the latent fatigue factor and the latent altered cognition factor (i.e., brain fog) each influenced the responses of 10 items. Clinically, however, fatigue has often been considered a manifestation of “mental fatigue” [39], symptomatic of underlying cognitive deficits [40]. Consequently, we specified a one factor model using CFA in which the 20 items loaded on one general factor. This permitted a comparison between the BIAFAC two-factor model of fatigue and brain fog with a potentially parsimonious one-factor model.

Various fit indices were used to assess how well these two models fit the data. Here, we used the Root Mean Squared Error of Approximation (RMSEA), the Comparative Fit Index (CFI), the Tucker-Lewis Index (TLI), and the standardized root-mean-square residual (SRMR). The recommended rules of thumb for acceptable model fit are RMSEA < .10 [41, 42], CFI and TLI > .90 [43, 44], and SRMR < .10 [42]. We further used a chi-square difference test [45] to examine whether the two-factor model outperformed the one-factor model in fitting the data. A non-significant test result would indicate no difference between the two models; hence, the more parsimonious one-factor model would be sufficient in interpreting the scale structure.

After we established the factor model for the FACs, a series of measurement invariance (MI) tests were examined [46, 47]. The MI test was conducted to examine whether we could assume that the FACs latent factors measured the same constructs between people with TBI and those without TBI. The MI was tested by conducting a series of nested CFA models with constraints to force specific parameters to be invariant between the TBI groups in the models. These constraints were ordered as follows: equal model structures (configural invariance), equal factor loadings (i.e., correlations between latent factors and the corresponding items) (metric invariance), equal intercepts (i.e., expected values of items if the mean of the latent factor equal to zero) (scalar invariance), and equal residuals (i.e., item variance which cannot be explained by latent factors; strict invariance) [48–51].

Conventionally, chi-square difference tests were used to compare two nested models and decide whether the less restricted model outperforms the next more restricted model (e.g., configural vs. metric). Given that the chi-square test is sensitive to large sample size (>300), we adopted the criteria for invariance developed through Chen’s simulation study [52]. Specifically, for testing factor loading invariance, a change from the less restricted model to the next more restricted model for CFI < -0.010, *Δ*RMSEA ≥ 0.015, and *Δ*RMR < 0.030 would indicate invariance. For testing the intercept or residual invariances, the model changes in CFI < -0.010, RMSEA ≥ 0.015, and SRMR < 0.010 would indicate invariance. The descriptive statistics and the Cronbach’s alpha were conducted using SPSS version 28 (IBM Corp., Armonk, NY). The omega composite reliability was estimated using the R package *MBESS* [53, 54]. CFA and measurement invariance tests were conducted using Mplus version 8.7.

## Results

**Fig 1:**
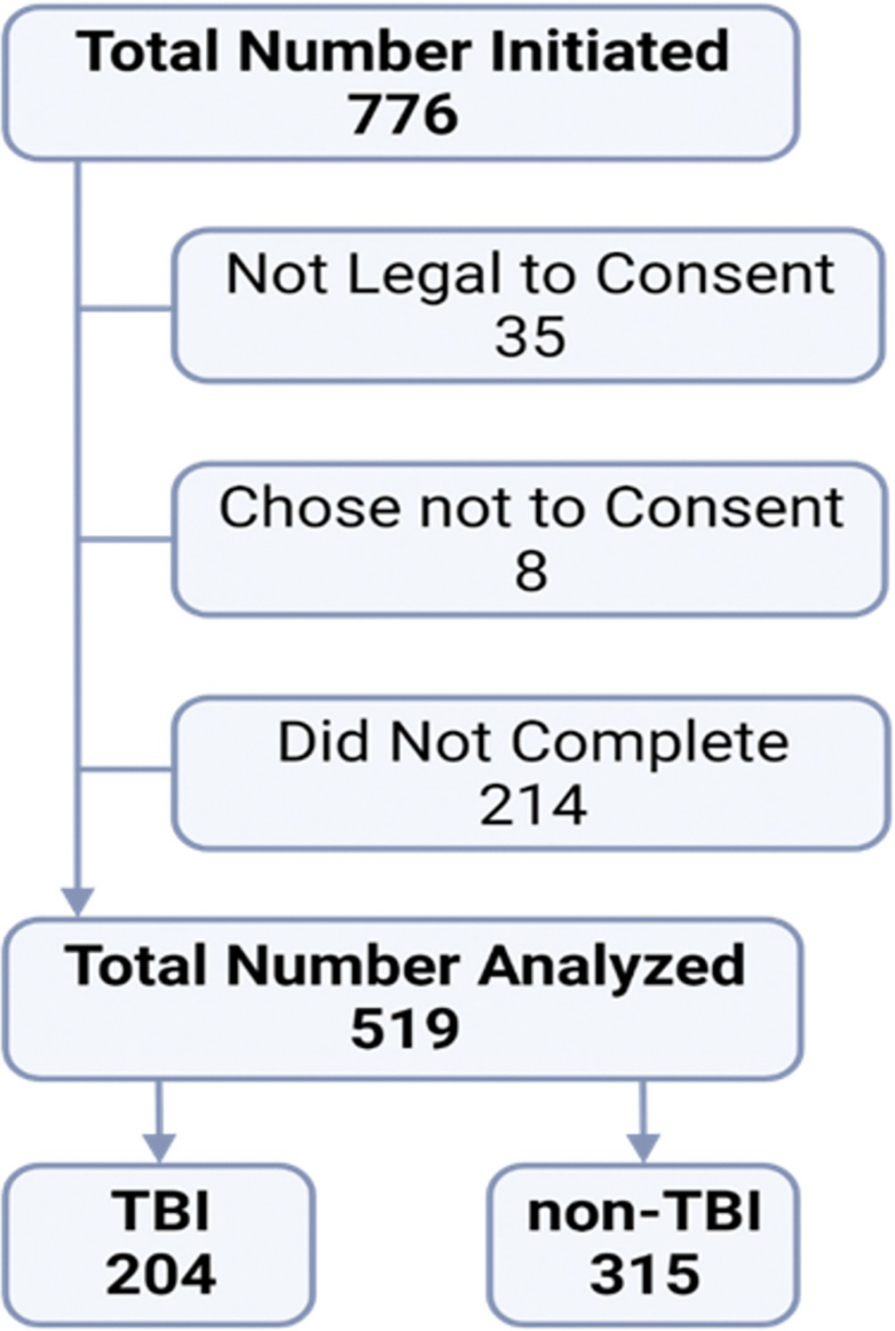
Flowchart of participant recruitment for data analysis.

A total of 776 participants initiated the digital FACs questionnaire (Fig 1). Upon digital screening, subjects were asked, “Have you been determined legally incapable of making decisions on your own behalf?” Thirty-five participants responded “yes” and were deemed incapable of providing consent and exited from the study without collecting further data. Eight individuals did not agree to consent, and an additional 214 were excluded because they did not complete the survey. This resulted in a final sample size of 519 consenting individuals who provided data suitable for analyses. The final number of participants is sufficient for our analyses to yield a stable factor structure.

The median time for subjects to complete the full online survey (including consent, demographics, and FACs questionnaire) was 4.5 minutes. Because participants that reported a history of TBI were prompted to respond to additional items (including the OSU-TBI) they took more time (median 6.2 minutes) than the non-TBI group (median 3.7 minutes).

The average age of the study participants was 40.23 years (*SD* = 16.28). The majority of participants identified as female (73%). A small percentage of the sample (12.3%) reported having had a positive COVID-19 diagnosis. Those reporting a TBI or concussion (39.3% of the sample, *n* = 204; mean age = 42 years) were significantly older than those in the non-TBI group (*n* = 315; mean age = 39.08; *p* < .05). There was no significant association between the distribution of individuals with TBI by gender (*p* = .105) or SARS-CoV-2 diagnosis (*p* = .293). Based on self-reported loss of consciousness (LOC) data reported on OSU TBI-ID, 75.5% of the participants had a mild TBI, consistent with the rate of mild TBI in the general population [29, 55, 56]. For the present study, severity of TBI was not critical to the development of the FACs, and this information will be examined in another report.

Descriptive statistics of the 20 items of the FACs are displayed in Table 1. Excluding item 15, all items had the minimum observations of 0 (item 15 had a minimum observation of 1) and the maximum observations of 100. Such results indicate that participants utilized the full score range designated in the questionnaire to represent their fatigue and cognition levels. The skewness values ranged from -0.39 to 1.12 and the kurtosis values ranged from -1.32 to 0.12, indicating that the responses from the 20 items followed normal distributions [57]. The range of the item means was 33.41 points, with the lowest mean of 24.60 for item 11 and the highest mean of 58.01 points for item 15, indicating that the FACs covered a wide range of item difficulty levels. The standard deviation of each item score ranged from 25.07 points to 32.99 points. Given normal distributions among the FACs items, we conclude that score differences of 50.14 points to 65.98 points covered around 68% of the participants in the sample. Hence, the FACs items can distinguish a wide range of responses.

**Table 1:** Item analyses of the 20 FACS items.

Item	Min	Max	Mean	SD	Skewness	Kurtosis
**Fatigue Scale**						
Q1: I felt fatigued	0	100	48.49	29.56	-0.02	-1.21
Q2: I felt alert*	0	100	39.30	25.07	0.38	-0.82
Q6: I felt worn out	0	100	53.22	30.59	-0.22	-1.16
Q7: I felt sluggish	0	100	43.80	30.20	0.16	-1.24
Q8: I felt run down	0	100	44.78	30.84	0.08	-1.28
Q10: I had the energy to do what I wanted to do*	0	100	49.14	27.97	0.06	-1.14
Q13: I had to force myself to get things done	0	100	48.66	32.15	-0.01	-1.30
Q15: I felt tired	1	100	58.01	29.49	-0.39	-1.00
Q17: I had to struggle to finish what I started to do	0	100	38.83	31.71	0.43	-1.17
Q20: I had problems feeling energetic no matter if I slept or napped	0	100	42.46	32.99	0.25	-1.32
**Altered Cognition Scale**						
Q3: I lost track of what I was going to say	0	100	41.45	30.16	0.31	-1.16
Q4: I was forgetful	0	100	39.92	30.17	0.46	-1.08
Q5: I had trouble concentrating	0	100	44.37	30.67	0.17	-1.27
Q9: I had trouble focusing on things I wanted to do	0	100	42.16	30.90	0.24	-1.23
Q11: I was easily confused	0	100	24.60	27.30	1.12	0.12
Q12: I felt “spaced out” like I was in a fog	0	100	30.66	30.49	0.78	-0.70
Q14: I was clear-headed*	0	100	44.55	29.31	0.16	-1.23
Q16: I didn’t process things as quickly or accurately as I should have	0	100	38.87	31.62	0.39	-1.21
Q18: I had trouble paying attention	0	100	40.18	30.73	0.30	-1.25
Q19: It was hard for me to make up my mind and reach a decision	0	100	34.53	31.74	0.61	-0.98

Notes. Min, minimum value observed; Max, maximum value observed; SD, standard deviation.

*Reverse coded before conducting item analyses

Cronbach’s alpha estimates of both the fatigue scale scores and the altered cognition scale scores were .95. The omega reliability values of both the fatigue and altered cognition scale scores were also .95. These measures indicate high reliability of both fatigue scale scores and cognition scale scores, suggesting researchers can use both scales for comparing individual differences [58].

To examine construct validity of the FACs, we evaluated the 20 items using both the one-factor and two-factor model. Descriptive statistics indicate that all FACs items followed a normal distribution. Hence, maximum likelihood estimation under the assumption that data were missing at random was employed. Modification indices were used for model refinement [59]. The one-factor model yielded poor fit to the data, *χ*^2^(170) = 2058.302, *p* < .001, CFI = 0.81, TLI = 0.79, RMSEA = 0.15, SRMR = 0.07. The two-factor model showed some room for improvement in model fit indices, *χ*^2^(169) = 1185.852, *p* < .001, CFI = 0.90, TLI = 0.88, RMSEA = 0.11, SRMR = 0.06, but it was an improvement over the one-factor model in terms of overall model fit indices. The chi-square difference test between these two models indicated that the two-factor model was statistically significantly better than the one-factor model, *χ*^2^(1) = 872.45, *p* < .001. These model results provide initial support for the BIAFAC two-factor model of fatigue and brain fog.

**Fig 2:**
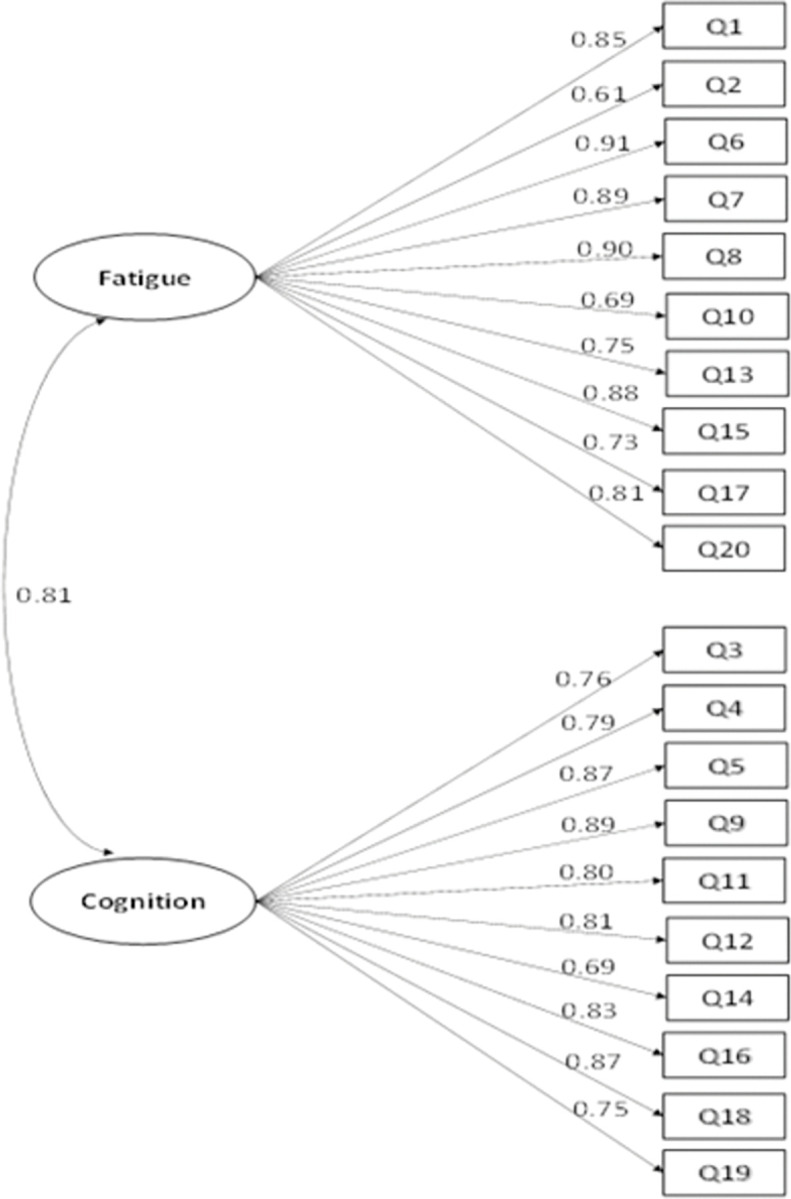
Standardized estimates of the two-factor model of the FACS. Note. The items’ residuals and the correlations of items’ residuals are omitted in this figure for clarity. This model has an acceptable fit, *χ*^2^(167) = 949.912, *p* < .001, CFI = 0.92, TLI = 0.91, RMSEA = 0.09, SRMR = 0.06.

After examining the modification indices, we added two item residual (i.e., item variance which cannot be explained by the latent factors) correlations to improve the model fit: The residual correlation between item 4 (*I was forgetful*) and item 3 (*I lost track of what I was going to say*), and that between item 13 (*I had to force myself to get things done*) and item 17 (*I had to struggle to finish what I started to do*). The revised model fit the data well: *χ*^2^(167) = 949.912, *p* < .001, CFI = 0.92, TLI = 0.91, RMSEA = 0.09, SRMR = 0.06 (Fig 2). The correlation between the fatigue factor and the altered cognition factor was 0.81. The standardized factor loadings (correlations between latent factors and the corresponding items) were close to or above 0.70 for all but three items. Such results indicated that around 50% of the variance in the items was explained by the theorized factors for most items. Overall, results from the CFA support using the two-factor model to depict the relationships among the FACs items.

Next, we examined the measurement invariance (MI) of the FACs between TBI and non-TBI groups. The FACs demonstrated metric, scalar, and strict invariance between TBI and non-TBI groups (see Table 2). Fit indices met Chen’s [52] criteria and indicated that more relaxed models did not perform significantly better than the model with more constraints. The MI results indicate that individuals with and without TBI perceived the FACs constructs similarly. Specifically, the FACs factor structures were the same between the two groups (configural invariance). Furthermore, regression-type analyses using the FACs scores were comparable between the two groups (metric invariance) [51, 60]. Finally, the comparison of the FACs scores between individuals of the TBI and non-TBI groups reflect their differences in the latent FACs levels (scalar and strict invariance) [61].

**Table 2:** Measurement invariance of FACS between TBI and non-TBI groups.

Model	*χ* ^2^	df	RMSEA	ΔRMSEA	CFI	ΔCFI	SRMR	ΔSRMR
**Configural**	1174.479	334	0.098	—	0.905	—	0.069	—
**Metric**	1226.415	352	0.098	< .001	0.901	-0.004	0.077	0.008
**Scalar**	1284.998	370	0.098	< .001	0.897	-0.004	0.076	-0.001
**Strict**	1334.867	390	0.097	-0.001	0.893	-0.004	0.077	0.001

Note. *χ*^2^ = chi-square; df = degrees of freedom; CFI = comparative fit index; ΔCFI = delta (change in) CFI; RMSEA = root mean square error of approximation; ΔSRMR = delta (change in) SRMR.

## Discussion

The results of the present study support the presumed two factor structure and item integrity of the FACs with a sample of participants with and without a self-reported history of TBI/concussion. Participants in both groups provided a wide range of responses, suggesting the response options of the eVAS were appropriate and useful. We demonstrate the function of the FACs in assessing this symptom cluster using a TBI model. Additional clinical and research implications of these data as they relate to TBI are explored in a separate manuscript. Importantly, the present study found adequate measurement invariance of the two factor FACs model between TBI and non-TBI participants. Testing the assumption of measurement invariance is a critical step in evaluating the psychometric properties of new assessments. By testing the measurement invariance of the FACs items, we established that the FACs framework can be measured equivalently by TBI and non-TBI participants. The scalar and residual invariance along with high reliability values (>.90) indicate that the fatigue and altered cognition scale scores effectively differentiate TBI and non-TBI groups.

The convenient digital format of the FACs can be completed by subjects in less than 4 minutes, provides a sensitive 100-point visual scale to report symptoms, and can be set to instantly self-score and report. Ideally, this format will prove useful in advancing our understanding and treatment of these co-occurring symptoms. The FACs can be easily adapted and configured for hand-held devices (e.g., laptop, iPad, smart phone), facilitating efficient and immediate clinical assessment of patient symptoms to improve treatment planning and monitoring. The FACs is designed to be sensitive to changes in altered cognition and central fatigue to document treatment response, which should minimize reliance on anecdotal patient reports and time-intensive cognitive tests. The FACs instrument provides clinicians with a mechanism to quantify the severity of brain fog and central fatigue symptoms to inform decisions about treatment, and the timing and degree of response of symptoms to treatment. This should enhance clinical management and monitoring of this symptom cluster.

Although fatigue and brain fog are often comorbid, our results suggest they also maintain measurable differences that justify distinct recognition and assessment. The central fatigue and brain fog in BIAFAC provides a working model to describe these co-occurring symptoms, both individually and cohesively using the FACs [2, 23]. Although our results support a two-factor model of central fatigue and brain fog, they do not resolve confusion about the overlap between these symptoms. Both are primarily based on patient self-report, lack clear definitions, and are often described in terms of other neuropsychological deficits (e.g., cognitive slowing, problems with working memory, sustained attention) [62, 63]. Nevertheless, the support for the two-factor model of central fatigue and brain fog in the present study implies that prevailing assumptions about mental fatigue–and accompanying measures–might merit empirical scrutiny to improve current definitions of the construct and facilitate greater precision in measurement. Further studies of the FACs could compare it with other self-report instruments currently used to assess mental fatigue [17, 39, 64] and brain fog [12].

Similarly, the FACs may be used to understand the complex associations observed between fatigue and brain fog with symptoms of depression, anxiety, and sleep disturbance [2, 17, 22, 23]. Patients with chronic health conditions who present with symptoms of central fatigue and brain fog may be seen as depressed and treated as such [2]. Specifically, studies comparing potential FACs symptom overlap with symptoms of depression and anxiety may help us better understand distinctions between these entities. This could help improve differential diagnoses for appropriate treatment strategies.

There are several limitations to this study. Unlike a traditional paper-based visual analog scale, the line length represented here is variable based on the size and orientation of the display screen. However, the intent of the eVAS is to serve as a visual representation of proportion and not absolute length. Those same principles of visually representing proportional agreement with the item cue are retained in this digital version and are automatically scored based on the digital sliding scale from 0 to 100 regardless of absolute line length. Our analysis suggests that the eVAS response format of the FACs exhibited psychometric properties we would expect from responses to these items administered on paper. The eVAS format of the FACs provides several advantages over paper administration, including automated scoring which removes potential operator bias or error, and a time-efficient capability to remotely query, score, and tally large numbers of participants.

Of the 776 individuals that initiated the survey, 214 failed to complete it (27.6%). Survey length could be a biasing factor for questionnaire completion when surveying a population for potential symptoms of fatigue and cognitive impairment (including attention difficulties). Further studies may be needed to optimize questionnaire length to ensure adequate participation, precision, and coverage while minimizing the potential impact of time and attention. The present study relied on a sample recruited through listservs and social media, and participants completed an online questionnaire. We selected TBI as our clinical population, given our ongoing clinical interest in TBI and the documented associations of co-occurring brain fog and fatigue among these individuals. We do not know if the two-factor structure of the FACs will replicate among other clinical conditions known to experience problems with these co-occurring symptoms. Future studies with clinical samples and documented medical conditions (e.g., PASC, multiple sclerosis) must be conducted to establish the validity, reliability, and utility of the FACs in clinical settings.

### Conclusions

Co-occurring brain fog and fatigue compromise the quality of life of many individuals with chronic health conditions, and present challenges for clinical assessment and treatment. A tool that provides sensitive, valid, and efficient assessment of these symptoms will benefit clinical practice and research. Our study provides initial psychometric support for the FACs, each of the two subscales and the individual items. Findings from the present study indicate that the FACs effectively assesses the symptom cluster of fatigue and altered cognition. The ability of the FACs to reproducibly quantify individual symptoms provides the potential for more specific diagnosis and treatment by clinicians across various clinical scenarios.

The eVAS format of the FACs presents unique possibilities to assess symptom severity and monitor patient response to treatment in clinical encounters and in patient surveys. The FACs can be rapidly and conveniently administered by tablet in clinical settings during patient down-time, or during patient-clinician interactions. For research purposes, the FACs instrument provides rapid and easy digital distribution (in person, email, text), low participant/patient burden to complete (< 4 minutes), high sensitivity (0-to-100-point VAS scale), and automated scoring when using equipped online software. Future research should further examine the psychometric properties of the instrument in comparisons with other established measures and other clinical populations. Its suitability for online survey research enhances the potential use of the FACs to obtain normative data about co-occurring brain fog and central fatigue experienced by individuals across a variety of chronic health conditions.

## Supporting information

S1 Dataset (XLSX)
